# Higher Levels of Urinary Thiocyanate, a Biomarker of Cruciferous Vegetable Intake, Were Associated With Lower Risks of Cardiovascular Disease and All-Cause Mortality Among Non-smoking Subjects

**DOI:** 10.3389/fnut.2022.919484

**Published:** 2022-07-05

**Authors:** Qiang Wang, Lei King, Pei Wang, Guanhua Jiang, Yue Huang, Changchang Dun, Jiawei Yin, Zhilei Shan, Jian Xu, Liegang Liu

**Affiliations:** ^1^Hubei Key Laboratory of Food Nutrition and Safety, Department of Nutrition and Food Hygiene, School of Public Health, Tongji Medical College, Huazhong University of Science and Technology, Wuhan, China; ^2^Ministry of Education (MOE) Key Lab of Environment and Health, School of Public Health, Tongji Medical College, Huazhong University of Science and Technology, Wuhan, China; ^3^Shenzhen Center for Chronic Disease Control, Shenzhen, China

**Keywords:** thiocyanate, biomarker, cruciferous vegetable, cardiovascular disease, mortality, National Health and Nutrition Examination Survey

## Abstract

**Background:**

Epidemiologic studies on cruciferous vegetable (CV) intake and cardiovascular disease (CVD) were inconclusive.

**Objective:**

To investigate the associations of urinary thiocyanate, a biomarker of CV intake, with CVD and all-cause mortality among non-smoking adults.

**Methods:**

This prospective cohort study comprised 10,489 non-smoking adults (weighted mean age, 46.8 years; 43.4% male) from the National Health and Nutrition Examination Survey 2001–2014. Non-smokers were defined as subjects with serum cotinine < 3 ng/mL. Urinary thiocyanate was measured with ion chromatography tandem mass spectrometry at baseline, and CVD and all-cause mortality were identified through linkage to National Death Index until December 31, 2015. Cox proportional hazards model was applied to estimate the hazard ratios (HRs) with 95% confidence intervals (CIs) for CVD and all-cause mortality.

**Results:**

A total of 800 deaths, of which 136 died of CVD, were ascertained within a median 7.8 years of follow-up. Urinary thiocyanate was positively correlated with total CV intake among non-smoking adults (*r*_*s*_ = 0.088, *P* < 0.001). Comparing extreme quartiles, the multivariate-adjusted HRs for CVD and all-cause mortality were 0.50 (95% CI: 0.29–0.85) and 0.75 (95% CI: 0.60–0.92), respectively. Each 1 μg/g creatinine increment of log-transformed urinary thiocyanate was associated with a 25% (HR: 0.75; 95% CI: 0.62–0.91) reduced CVD mortality risk and 12% (HR: 0.88; 95% CI: 0.81–0.96) reduced all-cause mortality risk. The documented inverse associations persisted in sensitivity analyses.

**Conclusion:**

Increased levels of urinary thiocyanate, a candidate biomarker of CV intake, were associated with low risks of CVD and total mortality among non-smoking adults. This prospective biomarker-based study provided further evidence to support the cardiovascular benefits of CVs.

## Introduction

Immense health and economic burdens are produced by cardiovascular disease (CVD) globally (1, 2). Despite the fact that fatalities attributable to CVD in United States declined during the 1980s to the 2010s, CVD remains the leading cause of death worldwide, and the global death toll of CVD was expected to exceed 23.6 million by 2030 (1). It was estimated by the American Heart Association in 2016 that nearly half of the American population would be affected by CVD to some extent and total costs of CVD would reach 1.1 trillion dollars by 2035 (1). Hence, much concern had been raised about developing effective strategies, such as healthy diets (3), to prevent CVD.

Cruciferous vegetables (CVs) are featured by their high glucosinolates contents (4) and had been recognized as part of healthy diet (5). Nonetheless, human studies on CV intake and CVD were inconclusive, with some prompting protective effects while others reporting no significant associations (Supplementary Table 1). Joshipura et al. (6) observed no significant association between CV intake and risk of ischemic CVD among 70,870 females from the Nurses’ Health Study (NHS) and 38,918 males from the Health Professionals’ Follow-Up Study (HPFS). However, a pooled analysis of two prospective cohort studies comprising 134,796 Chinese adults reported that higher CV intake was associated with a lower risk of mortality from CVD (7). Food frequency questionnaires (FFQs) were applied to estimate CV intake in previous studies (Supplementary Table 1), making the results susceptible to measurement error and misclassification. Hence, biomarker-based studies are anticipated to have a better understanding of the association between CV intake and risk of CVD.

Thiocyanate, a metabolite of cyanide from tobacco or glucosinolates from CVs (8), could be ubiquitously detected in urine samples (9). Elimination of thiocyanate occurs in the kidneys, and the half-life of thiocyanate is 3 days in individuals without renal insufficiency (8). Cigarette smoking makes considerable difference in the major source of thiocyanate, and thiocyanate primarily originates from tobacco for smokers and diet for non-smokers, respectively (10). Moreover, urinary thiocyanate levels vary between smokers and non-smokers, and smokers have much higher urinary thiocyanate measurements (11, 12). These facts provide a clue that urinary thiocyanate might be a biomarker of CV intake among non-smokers.

In this prospective cohort study of non-smoking adults, we aimed to investigate the associations of urinary thiocyanate with CVD and all-cause mortality. We hypothesized that higher levels of urinary thiocyanate, a biomarker of CV intake, may be associated with lower risks of CVD and all-cause mortality among non-smoking subjects.

## Materials and Methods

### Study Population

National Health and Nutrition Examinations Survey (NHANES) is a series of nationally representative surveys enrolling approximately 5,000 non-institutional civilians in the United States each year (13). This program had been approved by the National Center for Health Statistics (NCHS) Ethics Review Board and gained informed consent from participants. Details on the program were described elsewhere (13).

We used data from NHANES 2001–2014 cycles, from which a total of 69,236 participants with medical examination data were preliminarily selected. 33,004 adults were left after participants aged < 20 years, without mortality data, or having CVD at baseline were excluded. A total of 14,500 participants with complete urinary thiocyanate and creatinine and serum cotinine measurements were further identified. Benowitz et al. (14) recommended 3 ng/mL as the cut-off point to distinguish smokers and non-smokers based on a US nationally representative sample of 3,078 smokers and 13,078 non-smokers. Hence, 10,489 adults with serum cotinine < 3 ng/mL were identified as non-smokers and included in this prospective cohort study (Supplementary Figure 1).

### Urinary Thiocyanate Measurement

Spot urine specimens were collected into sterile 250-mL containers, following the instructions described in the NHANES Laboratory Procedures Manual (15). Urinary thiocyanate levels were determined with ion chromatography coupled with electrospray tandem mass spectrometry in the National Center for Environmental Health (16). Chromatographic separation of compounds was carried out in IonPac AS16 column with sodium hydroxide as the eluant (16). Details of laboratory methodology, quality control, and quality assurance were described elsewhere (16). The limit of detection (LOD) was 20 ng/mL for thiocyanate, and the detection limit divided by the square root of two was assigned as the corresponding value for the measurements below LOD.

### Outcomes Assessment

The outcomes of interest in our study were CVD and all-cause mortality, which were identified through linkage to the National Death Index until December 31, 2015 (17). Causes of death had been ascertained according to the International Classification of Diseases, Tenth Revision (ICD-10) by the NCHS. In the current study, CVD deaths were defined as deaths attributed to heart disease or cerebrovascular diseases (ICD-10 codes I00-I09, I11, I13, I20-I51, I60-I69). Follow-up duration was defined as the interval from the mobile examination center date to the date of death or December 31, 2015, whichever occurred first.

### Covariates Assessment

Demographic, socioeconomic, lifestyle, and dietary information was collected with questionnaires by trained interviewers. Race/ethnicity was categorized as non-Hispanic white, non-Hispanic black, Mexican American, and others (10, 18). Family poverty income ratio (PIR), a measure of family income, was classified into three categories (<1.3, 1.3- < 3.5, ≥ 3.5). Participants who had less than 12 alcohol drinks in their lifetime were classified as never drinkers; those who had at least 12 alcohol drinks but avoided alcohol in the past 12 months were defined as former drinkers; and individuals who drank alcohol in the past 12 months when surveyed were categorized as current drinkers. Physical activity was classified into never, moderate, and vigorous according to replies of respondents to the items related to daily, recreational, and sedentary activities. Vigorous activities were defined as activities that cause large increases in breathing or heart rate for at least 10 min, and moderate activities were defined as activities that cause small increases in breathing or heart rate for at least 10 min (19). Dietary information was obtained with 24-h dietary recall interviews by trained dietary interviewers, and total energy intake was calculated with the automated multiple pass method. Healthy Eating Index-2015 (HEI-2015), which comprises nine adequacy and four moderation components, was used to indicate overall diet quality (20). HEI-2015 ranged from 0 to 100, and a higher score indicated a better diet quality (20). A 139-item FFQ, which was developed from the validated National Cancer Institute Diet History Questionnaire (DHQ) (21), was added to NHANES 2003–2004 and 2005–2006 to obtain information on food and food group consumption patterns during the past year. The FFQ contained two items related to CV, “Did you eat broccoli?” and “Did you eat cauliflower?” and the answers ranged from “never” to “two or more times per day.” Total CV consumption (times/week) was calculated by summing the consumption of broccoli and cauliflower, two common *Brassica* species. The arithmetic mean of the upper and lower limits was used as the corresponding consumption. If the highest intake category interval was right-open (e.g., ≥ 7 times/week), the corresponding intake was set at 1.2 times the lower boundary (e.g., 8.4 times/week) (22). If the lowest intake category interval was left-open (e.g., < 1 time/week), the corresponding intake was set at half the upper boundary (e.g., 0.5 time/week) (23). Anthropometric information was collected by trained health technicians, and body mass index (BMI) was calculated as weight (kg) divided by height (m) squared. Cotinine, the metabolite of nicotine, has a half-life of 15–20 h in plasma, and was preferred as the biomarker of smoking in previous studies (14, 24, 25). Serum cotinine was measured with an isotope dilution-high performance liquid chromatography/atmospheric pressure chemical ionization tandem mass spectrometry (26). The detection limit of serum cotinine was 0.015 and 0.011 ng/mL was assigned as the corresponding value for the results below the detection limit (26). Second-hand smoking was defined as serum cotinine between LOD and 3-ng/mL cut-off point in this study. Blood pressure measurements were collected by examiners who had been certified through a training program with mercury sphygmomanometers. Hypertension was defined as systolic blood pressure (SBP) ≥ 140 mmHg, diastolic blood pressure (DBP) ≥ 90 mmHg, or currently taking prescribed medicine for hypertension (27). Urinary concentrations of creatinine were measured with an enzymatic method based on Jaffe rate reaction (28). Urinary iodine was measured with inductively coupled plasma-mass spectrometry (29) and categorized into low iodine excretion (<100 μg/L) and high iodine excretion (≥ 100 μg/L) according to the classification recommended by the World Health Organization (30).

### Statistical Analysis

Missing values of covariates were imputed with medians and missing indicators for continuous and categorical variables, respectively. Number and proportion of missing covariates were shown in Supplementary Table 2, and a total of 4,827 missing values were imputed. Urinary thiocyanate was divided by creatinine concentration to adjust for urine dilution, and log-transformed to alleviate the skewed distribution. Geometric means of urinary thiocyanate measurements according to population characteristics were calculated. Taking the complex, multistage, and probability sampling design of NHANES into account, we applied sampling weights and sample design variables in formal analyses.

Participants were categorized according to quartiles of urinary thiocyanate. Continuous variables were expressed with weighted means and standard errors, and categorical variables were expressed with numbers and weighted proportions. The baseline characteristics across thiocyanate quartiles were compared with linear regression for continuous variables and logistic regression for categorical variables. Partial Spearman correlation coefficient between urinary thiocyanate and total CV intake was calculated in a pilot study among NHANES 2005–2006. Cox proportional hazards regression model was applied to investigate the associations of urinary thiocyanate with risks of CVD and total mortality. The proportional hazards assumptions were tested with Schoenfeld residuals method, and no violation was observed. Confounders, including age (years, continuous), sex (male, female), race/ethnicity (non-Hispanic white, non-Hispanic black, Mexican American, others), secondhand smoking (yes, no), BMI (< 25, 25- < 30, ≥ 30 kg/m^2^), education attainment (under high school, high school, above high school), family PIR (< 1.3, 1.3- < 3.5, ≥ 3.5), alcohol consumption (never, former, current), physical activity (never, moderate, vigorous), total energy intake (kcal, continuous), HEI-2015 score (continuous), urinary iodine (< 100, ≥ 100 μg/L), and hypertension (yes, no), were adjusted in the multivariate models. The *P*-value for linear trend was calculated by introducing medians of quartiles as continuous variables into the model. We additionally calculated the multivariate-adjusted hazard ratios (HRs) with 95% confidence intervals (CIs) for CVD and total mortality associated with each 1 μg/g increment in log-transformed urinary thiocyanate. Restricted cubic splines with 3 knots at the 5th, 50th, and 95th percentiles of log-transformed urinary thiocyanate distribution were further plotted to examine the log-linear dose–response relationships between urinary thiocyanate and CVD and total mortality, and the reference value was set at the 10th percentile.

Stratified analyses by age (< 50 years, ≥ 50 years), sex (male, female), race/ethnicity (non-Hispanic white, others), obesity (yes, no), secondhand smoking (yes, no), current drinking (yes, no), hypertension (yes, no), and diet quality (lower, higher) were performed to examine whether these factors modified the association between urinary thiocyanate and CVD mortality. Potential interaction between urinary thiocyanate and stratification factor was evaluated by introducing a multiplicative term between urinary thiocyanate and stratification variable as continuous variables into the multivariate models, and testing whether the coefficient of the interaction term was equal to zero. Taking increased false positive in multiple hypothesis testing into account, we adjusted *P*-value with Bonferroni correction, and statistical significance was set at *P* < 0.006 (0.05/8 subgroups). We also calculated the false discovery rate (FDR) to identify as many significant interactions as possible while controlling a relatively low proportion of false positives, and FDR < 0.05 was considered as significant.

Moreover, several sensitivity analyses were conducted to evaluate the robustness of our results. First, we introduced urinary creatinine as a covariate into the multivariate models rather than divided thiocyanate by creatinine to adjust for urine dilution. Second, participants with daily energy intake < 500 or > 5,000 kcal were excluded to examine whether our results were sensitive to extreme daily energy intake. Third, we excluded the total vegetables component from the HEI-2015 score and precluded the adjustment of BMI to avoid potential over-adjustment, and introduced fasting plasma glucose into models. Fourth, dietary fiber, β-carotene, folate, vitamin K, total fruits, total dairy, and whole grains intake, rather than HEI-2015 score, were adjusted in multivariate models. Fifth, we adjusted SBP, DBP, and antihypertensive therapy rather than hypertension in models, considering the evidence that antihypertensive drug treatment could affect the cardiovascular outcomes (31, 32). Finally, multiple imputed data sets for missing covariates were generated under the missing-at-random assumption since single imputation did not reflect the uncertainty about the predictions of the missing values.

We used STATA 15.1 (StataCorp LLC, Texas, United States) and SAS 9.4 (SAS Institute, NC, United States) for statistical analysis. All tests were bilateral, and *P*-values lower than 0.05 were recognized as statistical significance unless otherwise stated.

## Results

### Characteristics of Study Population

This cohort comprised 10,489 non-smoking subjects (weighted mean age, 46.8 years; 43.4% male). The mean urinary thiocyanate was 1.28 mg/L. The geometric mean of urinary thiocyanate was 0.94 and 0.95 mg/g creatinine in never and secondhand smokers (Supplementary Table 3), respectively. Higher urinary thiocyanate levels were observed among female and non-Hispanic white participants (Supplementary Table 3). Non-smoking subjects with higher urinary thiocyanate levels were more likely to be current drinkers, have high family incomes and education attainments, exercise regularly, and have higher intakes of total vegetables, total dairy, whole grains, fiber, β-carotene, folate, and vitamin K (Table 1). There was no significant difference in serum cotinine levels across thiocyanate quartiles (*P* = 0.31). Urinary thiocyanate was positively correlated with total CV intake (*r*_*s*_ = 0.088, *P* < 0.001) in this non-smoking population (Supplementary Table 4).

**TABLE 1:** Characteristics of study population according to quartiles of urinary thiocyanate^a^.

Characteristics	Total	Quartiles of urinary thiocyanate				*P*-value
		1 (lowest)	2	3	4 (highest)	
No. of participants	10,489	2,623	2,624	2,620	2,622	
Age, years	46.8 ± 0.3	47.6 ± 0.5	45.6 ± 0.5	46.1 ± 0.5	47.8 ± 0.4	<0.001
Men, *n* (%)	4,519 (43.4)	1,296 (47.9)	1,148 (43.9)	1,045 (41.8)	1,030 (41.3)	<0.001
Race/ethnicity, *n* (%)						<0.001
Non-Hispanic white	4,640 (68.7)	809 (54.1)	1,034 (63.6)	1,254 (72.1)	1,543 (79.8)	
Non-Hispanic black	1,917 (9.7)	547 (12.6)	507 (11.2)	448 (9.1)	415 (7.0)	
Mexican American	2,212 (9.6)	730 (14.9)	588 (11.1)	528 (8.7)	366 (5.5)	
Others	1,720 (12.0)	537 (18.4)	495 (14.0)	390 (10.0)	298 (7.6)	
Family poverty income ratio, *n* (%)						<0.001
<1.3	2,448 (15.0)	758 (19.2)	646 (17.6)	539 (12.9)	505 (11.8)	
1.3- < 3.5	3,670 (32.3)	970 (35.4)	918 (32.6)	896 (30.5)	886 (31.5)	
>3.5	3,614 (47.1)	700 (39.9)	863 (43.7)	999 (51.4)	1,052 (51.0)	
Education attainment, *n* (%)						<0.001
Under high school	2,580 (14.4)	894 (21.2)	646 (15.3)	552 (11.8)	488 (11.2)	
High school	2,166 (20.5)	526 (20.7)	543 (20.5)	512 (19.8)	585 (21.1)	
Above high school	5,737 (65.0)	1,198 (57.8)	1,435 (64.2)	1,555 (68.4)	1,549 (67.7)	
Smoking status^b^, *n* (%)						0.10
Never	3,058 (30.7)	718 (29.4)	784 (30.6)	829 (33.0)	727 (29.6)	
Secondhand	7,431 (69.3)	1,905 (70.6)	1,840 (69.4)	1,791 (67.0)	1,895 (70.4)	
Alcohol consumption, *n* (%)						<0.001
Never	1,741 (13.3)	535 (17.5)	452 (14.8)	367 (11.3)	387 (11.0)	
Former	1,769 (14.0)	501 (16.0)	423 (13.9)	423 (13.5)	422 (13.2)	
Current	6,235 (66.4)	1,362 (58.6)	1,560 (64.8)	1,648 (69.0)	1,665 (70.6)	
Physical activity, *n* (%)						<0.001
Never	3,485 (26.3)	1,027 (31.7)	881 (27.7)	801 (24.3)	776 (23.1)	
Moderate	3,337 (33.0)	765 (30.0)	818 (33.1)	851 (33.2)	903 (34.9)	
Vigorous	3,583 (40.0)	800 (37.0)	904 (38.6)	948 (41.8)	931 (41.6)	
Hypertension, *n* (%)	3,437 (28.8)	952 (32.5)	800 (27.7)	790 (26.2)	895 (29.4)	0.003
BMI, kg/m^2^	28.7 ± 0.1	28.4 ± 0.2	28.8 ± 0.2	28.5 ± 0.2	28.9 ± 0.2	0.007
Urinary creatinine, mg/dL	115.5 ± 1.0	148.3 ± 2.3	129.5 ± 1.9	109.2 ± 1.8	87.0 ± 1.3	<0.001
Urinary thiocyanate, μg/L	1282.7 ± 23.1	458.4 ± 9.4	877.5 ± 12.7	1261.0 ± 20.2	2208.6 ± 43.6	<0.001
Urinary iodine, μg/L	331.6 ± 94.9	222.7 ± 8.7	268.1 ± 28.4	244.5 ± 29.2	538.1 ± 320.4	0.47
Serum cotinine, ng/mL	0.14 ± 0.01	0.13 ± 0.01	0.14 ± 0.01	0.14 ± 0.01	0.15 ± 0.01	0.31
Total energy intake, kcal	2133.7 ± 13.3	1995.2 ± 25.5	2121.0 ± 24.0	2179.8 ± 24.1	2199.2 ± 21.9	<0.001
HEI-2015 score	54.8 ± 0.2	54.3 ± 0.3	54.5 ± 0.3	55.0 ± 0.3	55.3 ± 0.4	0.04
Total fruits^c^	2.67 ± 0.03	2.83 ± 0.05	2.65 ± 0.05	2.67 ± 0.05	2.57 ± 0.05	<0.001
Total vegetables^c^	3.42 ± 0.02	3.27 ± 0.04	3.39 ± 0.03	3.45 ± 0.03	3.52 ± 0.03	<0.001
Total dairy^c^	5.50 ± 0.06	5.15 ± 0.08	5.40 ± 0.10	5.56 ± 0.09	5.75 ± 0.08	<0.001
Whole grains^c^	2.77 ± 0.05	2.56 ± 0.08	2.68 ± 0.08	2.86 ± 0.08	2.92 ± 0.09	<0.001
Dietary fiber, g	17.2 ± 0.2	16.3 ± 0.3	16.9 ± 0.3	17.6 ± 0.3	17.8 ± 0.3	<0.001
β-carotene, mg	2201.6 ± 58.8	1872.1 ± 88.9	2132.7 ± 95.3	2267.3 ± 126.2	2428.4 ± 114.1	<0.001
Folate, μg	420.1 ± 3.8	394.2 ± 6.8	412.7 ± 7.7	429.9 ± 5.8	435.3 ± 5.9	<0.001
Vitamin C, mg	90.1 ± 1.4	89.3 ± 2.3	89.6 ± 2.6	92.9 ± 2.3	88.6 ± 2.0	0.32
Vitamin K, mg	107.9 ± 2.4	94.5 ± 4.2	104.4 ± 3.6	107.7 ± 3.8	120.4 ± 4.4	<0.001

*^a^Continuous variables were expressed as weighted means and standard errors and categorical variables were expressed as numbers and weighted percentages. The sums of percentages may not reach 100%, owing to the rounding of decimals and missing values. Baseline characteristics across thiocyanate quartiles were compared with linear regression for continuous variables and logistic regression for categorical variables.*

*^b^Participants with serum cotinine ≤ 0.015 mg/dL and 0.015- < 3 mg/dL were considered as never and secondhand smokers, respectively.*

*^c^Total vegetables, total fruits, total dairy, and whole grains intake were estimated with HEI-2015 total vegetables, total fruits, total dairy, and whole grains components, respectively.*

*BMI, body mass index; HEI, Health Eating Index.*

### Associations of Urinary Thiocyanate With Cardiovascular Disease and All-Cause Mortality

**FIGURE 1:**
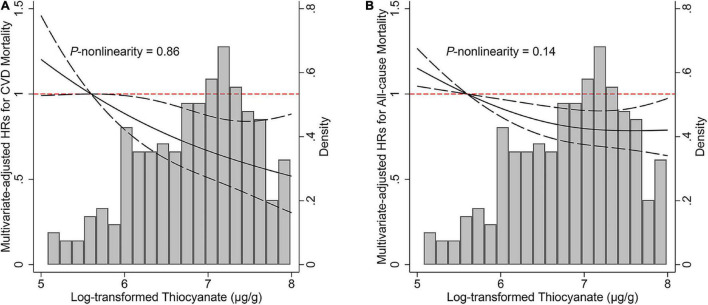
Associations of urinary thiocyanate levels with **(A)** cardiovascular disease and **(B)** all-cause mortality among non-smokers. Hazard ratio was represented by solid line and 95% confidence intervals were represented by dashes. Model was adjusted for age (years, continuous), sex (male, female), race/ethnicity (non-Hispanic white, non-Hispanic black, Mexican American, others), secondhand smoking (yes, no), body mass index (< 25, 25- < 30, ≥ 30 kg/m^2^), education attainment (under high school, high school, above high school), family poverty income ratio (< 1.3, 1.3- < 3.5, ≥ 3.5), alcohol consumption (never, former, current), physical activity (never, moderate, vigorous), total energy intake (kcal, continuous), Healthy Eating Index-2015 score (continuous), urinary iodine (< 100 μg/L, ≥ 100 μg/L), and hypertension (yes, no). CVD, cardiovascular disease; HR, hazard ratio.

During 78,095 person-years of observation (median follow-up, 7.8 years), a total of 800 deaths, of which 136 died of CVD, were ascertained. Comparing extreme thiocyanate quartiles, the multivariate-adjusted HRs for CVD and all-cause mortality were 0.50 (95% CI: 0.29–0.85, *P*-trend = 0.02) and 0.75 (95% CI: 0.60–0.92, *P*-trend = 0.009), respectively (Table 2). Inverse log-linear dose–response relationships between urinary thiocyanate and risks of CVD (*P*-non-linearity = 0.86) and total (*P*-non-linearity = 0.14) mortality were depicted in the restricted cubic splines (Figure 1). Each 1 μg/g creatinine increment of log-transformed urinary thiocyanate was associated with a 25% (HR: 0.75; 95% CI: 0.62–0.91) reduced risk of CVD mortality and 12% (HR: 0.88, 95% CI: 0.81–0.96) reduced risk of all-cause mortality (Table 2).

**TABLE 2:** Associations between urinary thiocyanate and cardiovascular disease and all-cause mortality among non-smoking adults.

	Quartiles of urinary thiocyanate				Continuous^c^	*P*-value for trend
	1 (*n* = 2,623)	2 (*n* = 2,624)	3 (*n* = 2,620)	4 (*n* = 2,622)		
Range, mg/g	≤0.50	0.50–0.89	0.89–1.51	>1.51		
**CVD mortality**						
No. of death	46	28	37	25		
Model 1^a^	1.00 (reference)	0.71 (0.42–1.22)	0.89 (0.57–1.40)	0.51 (0.30–.57)	0.77 (0.64–0.92)	0.02
Model 2^b^	1.00 (reference)	0.68 (0.40–1.15)	0.89 (0.56–1.43)	0.50 (0.29–0.85)	0.75 (0.62–0.91)	0.02
**All-cause mortality**						
No. of death	262	166	187	185		
Model 1^a^	1.00 (reference)	0.78 (0.61–0.99)	0.83 (0.68–1.02)	0.70 (0.57–0.86)	0.86 (0.80–0.94)	0.001
Model 2^b^	1.00 (reference)	0.77 (0.61–0.97)	0.86 (0.70–1.05)	0.75 (0.60–.92)	0.88 (0.81–0.96)	0.009

*^a^Model 1: adjusted for age (years, continuous), sex (male, female), race/ethnicity (non-Hispanic white, non-Hispanic black, Mexican American, others), secondhand smoking (yes, no).*

*^b^Model 2: further adjusted for body mass index (< 25, 25- < 30, ≥ 30 kg/m^2^), education attainment (under high school, high school, above high school), family poverty income ratio (< 1.3, 1.3- < 3.5, ≥ 3.5), alcohol consumption (never, former, current), physical activity (never, moderate, vigorous), total energy intake (kcal, continuous), Healthy Eating Index-2015 score (continuous), urinary iodine (< 100 μg/L, ≥ 100 μg/L), and hypertension (yes, no).*

*^c^Per 1 μg/g creatinine increment in log-transformed urinary thiocyanate.*

*CVD, cardiovascular disease; HR, hazard ratio.*

### Subgroup and Sensitivity Analyses

The association of urinary thiocyanate with CVD mortality stratified by several important confounders was depicted, and a stronger inverse association between urinary thiocyanate and CVD mortality was observed among non-Hispanic white participants (*P*-interaction = 0.002, FDR = 0.016) (Supplementary Figure 2). After directly introducing urinary creatinine as a covariate into the multivariate model to adjust for urine dilution, we consistently observed the inverse associations (Supplementary Table 5). Moreover, neither excluding extreme values of daily energy intake nor excluding the total vegetables component from HEI-2015 score distorted the documented inverse associations of thiocyanate exposure with CVD and all-cause mortality (Supplementary Tables 6, 7). The inverse associations persisted after excluding BMI from the multivariate models and further adjusting fasting plasma glucose in models (Supplementary Table 7). Comparing extreme quartiles, the HRs for CVD and all-cause mortality were 0.50 (95% CI: 0.29–0.85) and 0.75 (95% CI: 0.61–0.92) in the multivariate models where dietary fiber, β-carotene, folate, vitamin K, total fruits, total dairy, and whole grains intake were adjusted (Supplementary Table 8). In the multivariate model where SBP, DBP, and antihypertensive drug treatment were controlled, HRs (95% CIs) for CVD and all-cause mortality risk comparing extreme thiocyanate quartiles were 0.52 (0.30–0.91) and 0.75 (0.59–0.95), respectively (Supplementary Table 9). Similar results were observed after missing values of covariates were handled with multiple imputation (Supplementary Table 10).

## Discussion

In this prospective cohort of non-smoking adults, we observed inverse associations of urinary thiocyanate with CVD and total mortality. Comparing extreme quartiles, the multivariate-adjusted HRs for CVD and all-cause mortality were 0.50 (95% CI: 0.29–0.85) and 0.75 (95% CI: 0.60–0.92), respectively. Moreover, each 1 μg/g creatinine increment of log-transformed urinary thiocyanate was associated with a 25% (HR: 0.75; 95% CI: 0.62–0.91) reduced CVD mortality risk and 12% (HR: 0.88; 95% CI: 0.81–0.96) lower all-cause mortality risk.

To our knowledge, this cohort represents the first biomarker-based study to examine the associations of CV intake with CVD and all-cause mortality. Our findings were consistent with emerging studies that suggested the cardiovascular benefits of CVs (5, 7, 33, 34). An inverse association between CV intake and incident ischemic stroke was observed in a pooled analysis of two prospective cohort studies, which comprised 75,596 females in the NHS and 38,683 males in the HPFS (34). Zhang et al. (7) reported that higher CV intake was associated with a lower risk of mortality from CVD in two large Chinese prospective cohorts. Moreover, two recent meta-analyses of prospective studies provided the evidence of inverse association between CV intake and CVD (5, 35).

Estimating CV intake with quantitative biomarkers has received increasing attention (36, 37). Urinary isothiocyanate, another decomposed product of glucosinolates, had been applied in previous studies (36–38). A positive correlation between urinary isothiocyanate and self-reported CV intake (*r*_*s*_ = 0.1149, *P* < 0.0001) was observed in 3,589 females and 1,015 males from Shanghai (37). It should be noted that nearly half of isothiocyanates were eliminated after 2–4 h of CVs administration (38). The reproducibility, namely the correlation between samplings within the same individual on independent occasions (39), of a biomarker is determined by its half-life and the stability of individual intake of certain food/nutrient (39). Hence, the reproducibility of isothiocyanate might be poor in populations where CVs are less frequently or stably consumed. Compared with isothiocyanates, thiocyanates have a half-life of 3 days in healthy individuals (8). However, the validity of the urinary thiocyanate biomarker was questionable among smokers, and we observed no significant correlation between urinary thiocyanate and CV intake in smoking adults. In the current study, smokers had much higher urinary thiocyanate measurements than non-smokers in US adults (data not shown), consistent with previous studies (11, 12). A cross-sectional sample of 2027 females from NHANES 2003–2008 suggested that urinary thiocyanate levels among smokers were approximately five times higher than among non-smokers (11). After controlling environmental tobacco smoke, urinary thiocyanate was positively correlated with self-reported CV intake (*r*_*s*_ = 0.086, *P* < 0.001) among those with serum cotinine < 3 ng/mL in this study.

The underlying mechanisms of the documented inverse associations remain unclear, however, there are several possible explanations. First, CVs are important sources of dietary fiber, vitamins, and various phytochemicals, such as flavonoids (40) and sulforaphane (41), and these components are likely to act synergistically to enhance the cardiovascular benefits. There was convincing evidence of inverse association between dietary fiber and CVD mortality (42). A pooled analysis of 21 prospective studies provided evidence of inverse association between dietary vitamin K consumption and risk of coronary heart disease (43). A meta-analysis of 11 prospective cohort studies suggested that higher dietary flavonoid intake was associated with a lower risk of stroke (44). Moreover, sulforaphane had been suggested to protect against CVD due to its antioxidant and anti-inflammatory properties (45). Second, thiocyanate had been validated to play an important role in the host defense and protect cells against hypochlorous acid (HOCl), a powerful oxidant (8, 46). Hypothiocyanous acid, a product of hydrogen peroxide and thiocyanate catalyzed by peroxidases, is a potent antimicrobial agent and has the capability to cross the bacterial cell wall and inhibit the activity of glycolytic enzymes and urease (8, 46). Moreover, a previous *in vitro* study found that thiocyanate could exert influence on the extent and nature of HOCl-induced macrophage damage, and reported a protective effect of thiocyanate intervention on the development of chronic inflammation (47). Third, higher CV intake had been suggested to be associated with a lower risk of diabetes (48), a major risk factor for CVD (1).

Strengths of our study included the large sample of non-smoking adults and prospective and biomarker-based study design. Compared with interviews or self-reported FFQs, the quantitative biomarker could minimize the measurement error and misclassification introduced by biased recall, limited food items in questionnaires, and inaccurate portion-size estimation. Moreover, variability in glucosinolates contents across *Brassica* species, storage conditions, and preparing methods was allowed for with the measure of internalized exposure to *Brassica* thiocyanates.

Limitations of this prospective cohort study should be also acknowledged. First, we limited our analyses to those that were eligible for mortality linkage. Hence, internal validity of estimates derived from this cohort study is threatened due to follow-up bias (49). Second, urinary thiocyanate was measured with spot urine specimens at baseline. Hence, measurement error and potential misclassification might arouse due to variable hydration of participants and temporal variability. Single measurement of urinary thiocyanate might not reflect long-term exposure due to within-individual variability. Moreover, reproducibility of the urinary thiocyanate biomarker could not be examined with single measurements. Nonetheless, it was burdensome to collect repeated 24-h urine samples in large-scale surveys, and we performed conventional creatinine standardization as well as covariate adjustment to adjust for urine dilution and observed consistent results. Further longitudinal studies with repeated measurements are expected to validate the reproducibility of the biomarker and replicate our results. Third, although total CV intake was obtained from the FFQ developed from the validated DHQ, the FFQ only included two commonly consumed *Brassica* species. A previous cross-sectional study conducted in Korea, where residents consume large amounts of CVs, revealed that daily intake of thiocyanate through CVs varied across species (50). Moreover, portion size information was not collected with the FFQ, hence, we failed to obtain a more accurate estimate of CV intake. Hence, further validation studies with species and portion-size considered are needed. Fourth, low levels of thiocyanate could also be found in milk and cassava (10, 51, 52), hence, urinary thiocyanate is not perfectly specific for CVs in this non-smoking population. However, the inverse associations were not significantly altered by adjusting the total dairy adequacy component. Moreover, considering previously reported no significant associations of starchy vegetable intake with CVD and total mortality (53), the observed inverse associations in the current study may be attenuated. Finally, our results should be interpreted with caution due to residual confounding. Although we have adjusted total energy intake in multivariate models, consistent with the isocaloric diet/disease relationship of greatest interest, the standard multivariate model failed to completely adjust for confounding from common causes of dietary intake and composition (54). In addition, there are numerous nutrients and non-nutrients in foods due to the complexity of human diet, and residual confounding of other dietary factors could not be eliminated, although we have adjusted some nutrients and foods intake in multivariate models and observed unaltered results.

## Conclusion

This prospective cohort study suggested that higher levels of urinary thiocyanate, a biomarker of CV intake, were associated with lower risks of CVD and all-cause mortality among non-smoking adults. Our findings supported recommendations to increase CV consumption to promote cardiovascular health, and further studies are warranted to validate our results.

## Data Availability Statement

Publicly available datasets were analyzed in this study. These data can be found here: https://www.cdc.gov/nchs/nhanes/index.htm.

## Ethics Statement

The studies involving human participants were reviewed and approved by the NCHS Research Ethics Review Board. The patients/participants provided their written informed consent to participate in this study.

## Author Contributions

QW contributed to the conception and design of the study and drafted the manuscript. LK contributed to the analysis and interpretation of data. PW, GJ, and CD contributed to the eacquisition and interpretation of data. JY and ZS contributed to the study design. YH, JY, ZS, JX, and LL critically revised the manuscript for important intellectual content. LL was the guarantor of the work and had full access to the data underlying the article. All authors gave approval to the final manuscript and agreed to be accountable for all aspects of work ensuring integrity and accuracy.

## Conflict of Interest

The authors declare that the research was conducted in the absence of any commercial or financial relationships that could be construed as a potential conflict of interest.

## Publisher’s Note

All claims expressed in this article are solely those of the authors and do not necessarily represent those of their affiliated organizations, or those of the publisher, the editors and the reviewers. Any product that may be evaluated in this article, or claim that may be made by its manufacturer, is not guaranteed or endorsed by the publisher.
