# Supplementary material for: Higher Levels of Urinary Thiocyanate, a Biomarker of Cruciferous Vegetable Intake, Were Associated With Lower Risks of Cardiovascular Disease and All-Cause Mortality Among Non-smoking Subjects
Source: Front Nutr. 2022 Jul 5;9:919484. doi: 10.3389/fnut.2022.919484 (PMC9294399; doi:10.3389/fnut.2022.919484)
Supplement: Supplementary file 1 [file Data_Sheet_1.docx]

***Supplementary Material***

# Higher levels of urinary thiocyanate, a biomarker of cruciferous vegetable intake, was associated with lower risks of cardiovascular disease and all-cause mortality among non-smoking subjects

Qiang Wang, Lei King, Pei Wang, Guanhua Jiang, Yue Huang, Changchang Dun, Jiawei Yin, Zhilei Shan, Jian Xu, Liegang Liu

**Table of contents**

[Supplementary Table 1. Summary of previous findings on cruciferous vegetable intake and risk of cardiovascular disease. 3](#_Toc106199651)

[Supplementary Table 2. Number and proportion of missing covariates. 5](#_Toc106199652)

[Supplementary Table 3. Urinary creatinine-adjusted thiocyanate levels (mg/g creatinine), according to population characteristics. 6](#_Toc106199653)

[Supplementary Table 4. Partial Spearman correlation coefficients between urinary thiocyanate and broccoli, cauliflower, and total cruciferous vegetable intake among a subgroup of National Health and Nutrition Examination Survey 2005-2006 participants^a^. 7](#_Toc106199654)

[Supplementary Table 5. Associations between urinary thiocyanate and risks of cardiovascular disease and all-cause mortality among non-smoking adults, after introducing urinary creatinine as a covariate into models. 8](#_Toc106199655)

[Supplementary Table 6. Associations between urinary thiocyanate and risks of cardiovascular disease and all-cause mortality among non-smoking adults, after excluding extreme values of daily energy intake (< 500 kcal or > 5000 kcal). 9](#_Toc106199656)

[Supplementary Table 7. Associations between urinary thiocyanate and risks of cardiovascular disease and all-cause mortality among non-smoking adults, after excluding the total vegetables component from Healthy Eating Index-2015 score, excluding body mass index from models, and introducing fasting plasma glucose into models. 10](#_Toc106199657)

[Supplementary Table 8. Associations between urinary thiocyanate and risks of cardiovascular disease and all-cause mortality among non-smoking adults, after adjusting dietary fiber, *β*-carotene, folate, vitamin K, total fruits score, total dairy score, and whole grains score in the multivariate models. 12](#_Toc106199658)

[Supplementary Table 9. Associations between urinary thiocyanate and risks of cardiovascular disease and all-cause mortality among non-smoking adults, after adjusting systolic blood pressure, diastolic blood pressure, and antihypertensive drug treatment in the multivariate models. 13](#_Toc106199659)

[Supplementary Table 10. Associations between urinary thiocyanate and risks of cardiovascular disease and all-cause mortality among non-smoking adults, after applying multiple imputation under the missing-at-random to generate imputed datasets for missing covariates. 14](#_Toc106199660)

[Supplementary Figure 1. Flow diagram of participants selectionin National Health and Nutrition Examination Survey 2001-2014. 15](#_Toc106199661)

[Supplementary Figure 2. Adjusted hazard ratios for cardiovascular disease mortality associated with each 1 μg/g creatinine increment in log-transformed urinary thiocyanate in subgroups defined by age, sex, race/ethnicity, obesity, secondhand smoking, current drinking, hypertension, and diet quality among non-smokers. 16](#_Toc106199662)

[Supplementary References 17](#_Toc106199663)

## Supplementary Table 1. Summary of previous findings on cruciferous vegetable intake and risk of cardiovascular disease.

| **First author, year (ref)** | **Study population** | **Study type** | **Sample size** | **Sex** | **Age (years)** | **Dietary assessment method** | **Outcomes** | **Follow-up (years)** | **Results** |
| --- | --- | --- | --- | --- | --- | --- | --- | --- | --- |
| Hung, 2004 ([1](#_ENREF_1)) | NHS and HPFS | Prospective cohort study | 71910 females and 37725 males | M and F | NHS: 30-55, HPFS: 40-75 | FFQ | Incidence of fatal or nonfatal MI or stroke | NHS: 14, HPFS: 12 | No association |
| Zhang, 2011 ([2](#_ENREF_2)) | SWHS | Prospective cohort study | 74942 | F | 40-70 | FFQ | CVD mortality | 10.2 | HR (95% CI): 0.80 (0.72-0.89) (high vs. low) |
| Zhang, 2011 ([2](#_ENREF_2)) | SMHS | Prospective cohort study | 61500 | M | 40-74 | FFQ | CVD mortality | 4.6 | HR (95% CI): 0.73 (0.64-0.85) (high vs. low) |
| Blekkenhorst, 2017 ([3](#_ENREF_3)) | PLSAW | Prospective cohort study | 1226 | F | ≥ 70 | FFQ | Death relating to ASVD (ischemic heart disease, heart failure, cerebrovascular disease excluding hemorrhage, or peripheral heart disease) | 15 | HR (95%CI): 0.87 (0.81-0.94) (per 10 g/d increment) |
| Yu, 2013 ([4](#_ENREF_4)) | SWHS | Prospective cohort study | 67211 | F | 40-70 | FFQ | Incident CHD (nonfatal MI or fatal CHD) | 9.8 | No association |
| Yu, 2013 ([4](#_ENREF_4)) | SMHS | Prospective cohort study | 55474 | M | 40-75 | FFQ | Incident CHD (nonfatal MI or fatal CHD) | 5.4 | No association |
| Bhupathiraju, 2013 ([5](#_ENREF_5)) | NHS | Prospective cohort study | 71141 | F | 50 ± 7 | FFQ | Incident CHD (nonfatal MI or fatal CHD) | 24 | No association |
| Bhupathiraju, 2013 ([5](#_ENREF_5)) | HPFS | Prospective cohort study | 42135 | M | 53 ± 10 | FFQ | Incident CHD (nonfatal MI or fatal CHD) | 22 | No association |
| Joshipura, 1999 ([6](#_ENREF_6)) | NHS and HPFS | Prospective cohort study | 75596 females and 38683 males | M and F | NHS: 34-59,  HPFS: 40-75 | FFQ | Incident ischemic stroke | NHS: 14, HPFS: 8 | RR (95% CI): 0.71 (0.55-0.93) (high vs. low) |
| Joshipura, 2009 ([7](#_ENREF_7)) | NHS and HPFS | Prospective cohort study | 70870 females and 38918 males | M and F | NHS: 30-55, HPFS: 40-75 | FFQ | Incident ischemic CVD (fatal and nonfatal MI and ischemic stroke) | NHS: 16, HPFS: 14 | No association |
| Johnsen, 2003 ([8](#_ENREF_8)) | DDCHS | Prospective cohort study | 54506 | M and F | 50-64 | FFQ | Incident ischemic stroke (ischemic infraction, intracerebral hemorrhage or subarachnoid hemorrhage) | 3.09 | No association |
| Mizrahi, 2009 ([9](#_ENREF_9)) | FMCHES | Prospective cohort study | 3932 | M and F | 40-74 | FFQ | Incident ischemic stroke | 24 | RR (95% CI): 0.67 (0.49-0.92) (high vs. low) |
| Mizrahi, 2009 ([9](#_ENREF_9)) | FMCHES | Prospective cohort study | 3932 | M and F | 40-74 | FFQ | Incident intracerebral hemorrhage | 24 | RR (95% CI): 0.49 (0.25-0.98) (high vs. low) |
| Larsson, 2013 ([10](#_ENREF_10)) | SMC and COSM | Prospective cohort study | 74961 | M and F | 45-83 | FFQ | Stroke (cerebral infraction, hemorrhagic stroke or unspecified stroke) | 10.2 | No association |
| Wang, 2016 ([11](#_ENREF_11)) | Linxian NIT | Prospective cohort study | 2445 | M and F | 40-69 | FFQ | Heart disease mortality | 26 | No association |
| Wang, 2016 ([11](#_ENREF_11)) | Linxian NIT | Prospective cohort study | 2445 | M and F | 40-69 | FFQ | Stroke mortality | 26 | No association |
| Lockheart, 2007 ([12](#_ENREF_12)) | Norweigian men and postmenopausal women | Case-control study | 211 (106 cases and 105 controls) | M and F | 45-75 | FFQ | MI | - | OR (95% CI): 0.66 (0.47-0.93) (per SD increment) |

Abbreviations: ASVD, atherosclerotic vascular disease; CHD, coronary heart disease; COSM, Cohort of Swedish Men; CVD, cardiovascular disease; DDCHS, Danish Diet, Cancer, and Healthy Study; FFQ, food frequency questionnaire; FMCHES, Finnish Mobile Health Examination Survey; HPFS, Health Professionals Follow-up Study; HR, hazard ratio; MI, myocardial infraction; NHS, Nurses’ Health Study; NIT, Nutritional Intervention Trials; PLSAW, Perth Longitudinal Study of Ageing Women; SD, standard deviation; SMC, Swedish Mammography Cohort; SMHS, Shanghai Men’s Health Study; SWHS, Shanghai Women’s Health Study.

## Supplementary Table 2. Number and proportion of missing covariates.

| **Covariates** | **No. of missing values** | **Missing proportion (%)** |
| --- | --- | --- |
| Age | 0 | 0 |
| Sex | 0 | 0 |
| Race/ethnicity | 0 | 0 |
| Secondhand smoking | 0 | 0 |
| Body mass index | 113 | 1.1 |
| Education attainment | 6 | 0.06 |
| Family PIR | 757 | 7.2 |
| Alcohol consumption | 744 | 7.1 |
| Physical activity | 84 | 0.8 |
| Total energy intake | 459 | 4.4 |
| HEI-2015 score | 459 | 4.4 |
| Urinary iodine | 1923 | 18.3 |
| Hypertension | 282 | 2.7 |

Abbreviations: HEI, Health Eating Index; PIR, poverty income ratio.

## Supplementary Table 3. Urinary creatinine-adjusted thiocyanate levels (mg/g creatinine), according to population characteristics.

| **Characteristics** | **N** | **GMs (95% CIs)** |
| --- | --- | --- |
| Overall | 10489 | 0.95 (0.92-0.98) |
| Sex |  |  |
| Male | 4519 | 0.91 (0.87-0.95) |
| Female | 5970 | 0.98 (0.95-1.02) |
| Race/ethnicity |  |  |
| Non-Hispanic white | 4640 | 1.07 (1.02-1.11) |
| Non-Hispanic black | 1917 | 0.77 (0.74-0.80) |
| Mexican American | 2212 | 0.71 (0.68-0.74) |
| Others | 1720 | 0.72 (0.68-0.77) |
| Education attainment |  |  |
| Under high school | 2580 | 0.76 (0.72-0.81) |
| High school | 2166 | 0.96 (0.90-1.01) |
| Above high school | 5737 | 1.00 (0.96-1.03) |
| Family poverty income ratio |  |  |
| < 1.3 | 2448 | 0.80 (0.76-0.85) |
| 1.3-<3.5 | 3670 | 0.92 (0.88-0.97) |
| > 3.5 | 3614 | 1.02 (0.98-1.07) |
| Smoking status^a^ |  |  |
| Never | 3058 | 0.94 (0.91-0.98) |
| Secondhand | 7431 | 0.95 (0.91-0.99) |
| Alcohol consumption |  |  |
| Never | 1741 | 0.81 (0.76-0.87) |
| Former | 1769 | 0.88 (0.84-0.93) |
| Current | 6235 | 1.09 (0.97-1.05) |
| Physical activity |  |  |
| Never | 3485 | 0.86 (0.81-0.92) |
| Moderate | 3337 | 0.99 (0.95-1.03) |
| Vigorous | 3583 | 0.98 (0.95-1.02) |
| BMI, kg/m^2^ |  |  |
| < 25 | 2943 | 0.93 (0.90-0.96) |
| 25-<30 | 3610 | 0.94 (0.90-0.98) |
| ≥ 30 | 3823 | 0.98 (0.93-1.02) |
| Urinary iodine, μg/L |  |  |
| < 100 | 2797 | 1.08 (1.03-1.12) |
| ≥ 100 | 5769 | 0.91 (0.88-0.94) |

^a^ Participants with serum cotinine ≤ 0.015 mg/dL and 0.015-<3 mg/dL were considered as never and secondhand smokers, respectively.

Abbreviations: BMI, body mass index; GM, geometric mean

## Supplementary Table 4. Partial Spearman correlation coefficients between urinary thiocyanate and broccoli, cauliflower, and total cruciferous vegetable intake among a subgroup of National Health and Nutrition Examination Survey 2005-2006 participants^a^.

|  | **Partial Spearman correlation coefficients** | | | |
| --- | --- | --- | --- | --- |
|  | **Model 1^b^** | ***P*-value** | **Model 2^c^** | ***P*-value** |
| Non-smoking^d^ subjects (*n*=2192) |  |  |  |  |
| Broccoli (times/week)^e^ | 0.080 | < 0.001 | 0.082 | < 0.001 |
| Cauliflower (times/week)^e^ | 0.062 | 0.004 | 0.062 | 0.004 |
| Total cruciferous vegetable (times/week)^e^ | 0.086 | < 0.001 | 0.088 | < 0.001 |
| Smoking^d^ subjects (*n*=774) |  |  |  |  |
| Broccoli (times/week)^e^ | 0.053 | 0.14 | 0.066 | 0.066 |
| Cauliflower (times/week)^e^ | 0.040 | 0.26 | 0.062 | 0.084 |
| Total cruciferous vegetable (times/week)^e^ | 0.049 | 0.17 | 0.070 | 0.054 |

^a^ Participants aged ≥ 20 years, and having complete data on body mass index, serum cotinine, urinary creatinine and thiocyanate, and intakes of broccoli and cauliflower were included.

^b^ Adjusted for age, sex, race/ethnicity, body mass index.

^c^ Further adjusted for serum cotinine.

^d^ Non-smoking subjects were defined as participants with serum cotinine < 3 ng/mL, and smoking subjects were defined as participants with serum cotinine ≥ 3 ng/mL.

^e^ Intakes of broccoli, cauliflower, and total cruciferous vegetable were obtained from the NHANES Food Propensity Questionnaire. Total cruciferous vegetable intake was calculated by summing the consumption of broccoli and cauliflower. The arithmetic mean of the upper and lower limits was used as the corresponding consumption. If the highest intake category interval was right-open, the corresponding intake was set at 1.2 times the lower boundary. If the lowest intake category interval was left-open, the corresponding intake was set at half the upper boundary.

## Supplementary Table 5. Associations between urinary thiocyanate and risks of cardiovascular disease and all-cause mortality among non-smoking adults, after introducing urinary creatinine as a covariate into models.

|  | **Quartiles of urinary thiocyanate** | | | | **Continuous^c^** | ***P*-value for trend** |
| --- | --- | --- | --- | --- | --- | --- |
|  | **1 (*n*=2627)** | **2 (*n*=2618)** | **3 (*n*=2626)** | **4 (*n*=2618)** |  |  |
| Range, mg/L | ≤ 0.45 | 0.45-0.85 | 0.85-1.50 | > 1.51 |  |  |
| CVD mortality |  |  |  |  |  |  |
| No. of death | 53 | 30 | 29 | 24 |  |  |
| Model 1^a^ | 1.00 (reference) | 0.61 (0.37-1.00) | 0.56 (0.32-0.97) | 0.54 (0.33-0.87) | 0.73 (0.62-0.85) | 0.01 |
| Model 2^b^ | 1.00 (reference) | 0.60 (0.35-1.00) | 0.55 (0.30-1.00) | 0.55 (0.32-0.94) | 0.72 (0.60-0.87) | 0.02 |
| All-cause mortality |  |  |  |  |  |  |
| No. of death | 266 | 199 | 183 | 152 |  |  |
| Model 1^a^ | 1.00 (reference) | 0.74 (0.57-0.97) | 0.70 (0.55-0.90) | 0.72 (0.56-0.93) | 0.85 (0.76-0.94) | 0.006 |
| Model 2^b^ | 1.00 (reference) | 0.75 (0.58-0.97) | 0.69 (0.54-0.88) | 0.72 (0.56-0.93) | 0.84 (0.76-0.94) | 0.005 |

^a^ Model 1: adjusted for urinary creatinine (mg/dL, continuous), age (years, continuous), sex (male, female), race/ethnicity (non-Hispanic white, non-Hispanic black, Mexican American, others), secondhand smoking (yes, no).

^b^ Model 2: further adjusted for body mass index (< 25, 25-<30, ≥ 30 kg/m^2^), education attainment (under high school, high school, above high school), family poverty income ratio (< 1.3, 1.3-<3.5, ≥ 3.5), alcohol consumption (never, former, current), physical activity (never, moderate, vigorous), total energy intake (kcal, continuous), Healthy Eating Index-2015 score (continuous), urinary iodine (< 100, ≥ 100 μg/L), and hypertension (yes, no).

^c^ Per 1 μg/L increment in log-transformed urinary thiocyanate.

Abbreviations: CVD, cardiovascular disease; HR, hazard ratio.

## Supplementary Table 6. Associations between urinary thiocyanate and risks of cardiovascular disease and all-cause mortality among non-smoking adults, after excluding extreme values of daily energy intake (< 500 kcal or > 5000 kcal).

|  | **Quartiles of urinary thiocyanate** | | | | **Continuous^c^** | ***P*-value for trend** |
| --- | --- | --- | --- | --- | --- | --- |
|  | **1 (*n*=2582)** | **2 (*n*=2583)** | **3 (*n*=2581)** | **4 (*n*=2582)** |  |  |
| Range, mg/g | ≤ 0.50 | 0.50-0.89 | 0.89-1.51 | > 1.51 |  |  |
| CVD mortality |  |  |  |  |  |  |
| No. of death | 44 | 27 | 36 | 25 |  |  |
| Model 1^a^ | 1.00 (reference) | 0.66 (0.39-1.13) | 0.92 (0.58-1.46) | 0.53 (0.31-0.90) | 0.78 (0.65-0.94) | 0.04 |
| Model 2^b^ | 1.00 (reference) | 0.63 (0.38-1.04) | 0.90 (0.55-1.46) | 0.51 (0.30-0.86) | 0.76 (0.62-0.94) | 0.03 |
| All-cause mortality |  |  |  |  |  |  |
| No. of death | 257 | 162 | 185 | 184 |  |  |
| Model 1^a^ | 1.00 (reference) | 0.77 (0.60-0.98) | 0.84 (0.69-1.03) | 0.72 (0.58-0.88) | 0.87 (0.80-0.95) | 0.003 |
| Model 2^b^ | 1.00 (reference) | 0.76 (0.60-0.96) | 0.86 (0.70-1.06) | 0.76 (0.62-0.95) | 0.89 (0.82-0.98) | 0.02 |

^a^ Model 1: adjusted for age (years, continuous), sex (male, female), race/ethnicity (non-Hispanic white, non-Hispanic black, Mexican American, others), secondhand smoking (yes, no).

^b^ Model 2: further adjusted for body mass index (< 25, 25-<30, ≥ 30 kg/m^2^), education attainment (under high school, high school, above high school), family poverty income ratio (< 1.3, 1.3-<3.5, ≥ 3.5), alcohol consumption (never, former, current), physical activity (never, moderate, vigorous), total energy intake (kcal, continuous), Healthy Eating Index-2015 score (continuous), urinary iodine (< 100, ≥ 100 μg/L), and hypertension (yes, no).

^c^ Per 1 μg/g creatinine increment in log-transformed urinary thiocyanate.

Abbreviations: CVD, cardiovascular disease; HR, hazard ratio.

## Supplementary Table 7. Associations between urinary thiocyanate and risks of cardiovascular disease and all-cause mortality among non-smoking adults, after excluding the total vegetables component from Healthy Eating Index-2015 score, excluding body mass index from models, and introducing fasting plasma glucose into models.

|  | **Quartiles of urinary thiocyanate** | | | | **Continuous^d^** | ***P*-value for trend** |
| --- | --- | --- | --- | --- | --- | --- |
|  | **1 (*n*=2623)** | **2 (*n*=2624)** | **3 (*n*=2620)** | **4 (*n*=2622)** |  |  |
| Range, mg/g | ≤ 0.50 | 0.50-0.89 | 0.89-1.51 | > 1.51 |  |  |
| CVD mortality |  |  |  |  |  |  |
| No. of death | 46 | 28 | 37 | 25 |  |  |
| Model 1^a^ | 1.00 (reference) | 0.71 (0.42-1.22) | 0.89 (0.57-1.40) | 0.51 (0.30-0.87) | 0.77 (0.64-0.92) | 0.02 |
| Model 2^b^ | 1.00 (reference) | 0.68 (0.40-1.15) | 0.89 (0.56-1.43) | 0.50 (0.29-0.85) | 0.75 (0.62-0.91) | 0.02 |
| Model 3^c^ | 1.00 (reference) | 0.71 (0.42-1.20) | 0.90 (0.56-1.44) | 0.49 (0.29-0.84) | 0.75 (0.62-0.91) | 0.02 |
| Model 4^d^ | 1.00 (reference) | 0.68 (0.40-1.15) | 0.90 (0.56-1.44) | 0.50 (0.29-0.85) | 0.76 (0.63-0.92) | 0.02 |
| All-cause mortality |  |  |  |  |  |  |
| No. of death | 262 | 166 | 187 | 185 |  |  |
| Model 1^a^ | 1.00 (reference) | 0.78 (0.61-0.99) | 0.83 (0.68-1.02) | 0.70 (0.57-0.86) | 0.86 (0.80-0.94) | 0.001 |
| Model 2^b^ | 1.00 (reference) | 0.77 (0.61-0.97) | 0.86 (0.70-1.05) | 0.74 (0.60-0.92) | 0.88 (0.81-0.96) | 0.008 |
| Model 3^c^ | 1.00 (reference) | 0.79 (0.63-0.99) | 0.87 (0.71-1.07) | 0.74 (0.60-0.92) | 0.88 (0.81-0.96) | 0.008 |
| Model 4^d^ | 1.00 (reference) | 0.77 (0.61-0.97) | 0.86 (0.70-1.06) | 0.75 (0.60-0.92) | 0.89 (0.81-0.97) | 0.009 |

^a^ Model 1: adjusted for age (years, continuous), sex (male, female), race/ethnicity (non-Hispanic white, non-Hispanic black, Mexican American, others), secondhand smoking (yes, no).

^b^ Model 2: model 1 + body mass index (< 25, 25-<30, ≥ 30 kg/m^2^), education attainment (under high school, high school, above high school), family poverty income ratio (< 1.3, 1.3-<3.5, ≥ 3.5), alcohol consumption (never, former, current), physical activity (never, moderate, vigorous), total energy intake (kcal, continuous), modified Healthy Eating Index-2015 score (continuous), urinary iodine (< 100, ≥ 100 μg/L), and hypertension (yes, no).

^c^ Model 3: model 1 + education attainment (under high school, high school, above high school), family poverty income ratio (< 1.3, 1.3-<3.5, ≥ 3.5), alcohol consumption (never, former, current), physical activity (never, moderate, vigorous), total energy intake (kcal, continuous), Healthy Eating Index-2015 score (continuous), urinary iodine (< 100, ≥ 100 μg/L), and hypertension (yes, no).

^d^ Model 4: model 1 + body mass index (< 25, 25-<30, ≥ 30 kg/m^2^), education attainment (under high school, high school, above high school), family poverty income ratio (< 1.3, 1.3-<3.5, ≥ 3.5), alcohol consumption (never, former, current), physical activity (never, moderate, vigorous), total energy intake (kcal, continuous), Healthy Eating Index-2015 score (continuous), urinary iodine (< 100, ≥ 100 μg/L), fasting plasma glucose (mg/dL, continuous), and hypertension (yes, no).

^d^ Per 1 μg/g creatinine increment in log-transformed urinary thiocyanate.

Abbreviations: CVD, cardiovascular disease; HR, hazard ratio.

## Supplementary Table 8. Associations between urinary thiocyanate and risks of cardiovascular disease and all-cause mortality among non-smoking adults, after adjusting dietary fiber, *β*-carotene, folate, vitamin K, total fruits score, total dairy score, and whole grains score in the multivariate models.

|  | **Quartiles of urinary thiocyanate** | | | | **Continuous^c^** | ***P*-value for trend** |
| --- | --- | --- | --- | --- | --- | --- |
|  | **1 (*n*=2623)** | **2 (*n*=2624)** | **3 (*n*=2620)** | **4 (*n*=2622)** |  |  |
| Range, mg/g | ≤ 0.50 | 0.50-0.89 | 0.89-1.51 | > 1.51 |  |  |
| CVD mortality |  |  |  |  |  |  |
| No. of death | 46 | 28 | 37 | 25 |  |  |
| Model 1^a^ | 1.00 (reference) | 0.71 (0.42-1.22) | 0.89 (0.57-1.40) | 0.51 (0.30-0.87) | 0.77 (0.64-0.92) | 0.02 |
| Model 2^b^ | 1.00 (reference) | 0.69 (0.41-1.15) | 0.88 (0.55-1.43) | 0.50 (0.29-0.85) | 0.75 (0.62-0.92) | 0.02 |
| All-cause mortality |  |  |  |  |  |  |
| No. of death | 262 | 166 | 187 | 185 |  |  |
| Model 1^a^ | 1.00 (reference) | 0.78 (0.61-0.99) | 0.83 (0.68-1.02) | 0.70 (0.57-0.86) | 0.86 (0.80-0.94) | 0.001 |
| Model 2^b^ | 1.00 (reference) | 0.77 (0.61-0.97) | 0.85 (0.69-1.05) | 0.75 (0.61-0.92) | 0.89 (0.82-0.96) | 0.008 |

^a^ Model 1: adjusted for age (years, continuous), sex (male, female), race/ethnicity (non-Hispanic white, non-Hispanic black, Mexican American, others), secondhand smoking (yes, no).

^b^ Model 2: further adjusted for body mass index (< 25, 25-<30, ≥ 30 kg/m^2^), education attainment (under high school, high school, above high school), family poverty income ratio (< 1.3, 1.3-<3.5, ≥ 3.5), alcohol consumption (never, former, current), physical activity (never, moderate, vigorous), total energy intake (kcal, continuous), dietary fiber (g, continuous), *β*-carotene (mg, continuous), folate (μg, continuous), vitamin K (μg, continuous), total fruits score (continuous), total dairy score (continuous), whole grains score (continuous), urinary iodine (< 100, ≥ 100 μg/L), and hypertension (yes, no).

^c^ Per 1 μg/g creatinine increment in log-transformed urinary thiocyanate.

Abbreviations: CVD, cardiovascular disease; HR, hazard ratio.

## Supplementary Table 9. Associations between urinary thiocyanate and risks of cardiovascular disease and all-cause mortality among non-smoking adults, after adjusting systolic blood pressure, diastolic blood pressure, and antihypertensive drug treatment in the multivariate models.

|  | **Quartiles of urinary thiocyanate** | | | | **Continuous^c^** | ***P*-value for trend** |
| --- | --- | --- | --- | --- | --- | --- |
|  | **1 (*n*=2623)** | **2 (*n*=2624)** | **3 (*n*=2620)** | **4 (*n*=2622)** |  |  |
| Range, mg/g | ≤ 0.50 | 0.50-0.89 | 0.89-1.51 | > 1.51 |  |  |
| CVD mortality |  |  |  |  |  |  |
| No. of death | 46 | 28 | 37 | 25 |  |  |
| Model 1^a^ | 1.00 (reference) | 0.71 (0.42-1.22) | 0.89 (0.57-1.40) | 0.51 (0.30-0.57) | 0.77 (0.64-0.92) | 0.02 |
| Model 2^b^ | 1.00 (reference) | 0.71 (0.42-1.22) | 0.92 (0.59-1.45) | 0.52 (0.30-0.91) | 0.65 (0.31-0.91) | 0.04 |
| All-cause mortality |  |  |  |  |  |  |
| No. of death | 262 | 166 | 187 | 185 |  |  |
| Model 1^a^ | 1.00 (reference) | 0.78 (0.61-0.99) | 0.83 (0.68-1.02) | 0.70 (0.57-0.86) | 0.86 (0.80-0.94) | 0.001 |
| Model 2^b^ | 1.00 (reference) | 0.78 (0.60-1.02) | 0.88 (0.71-1.09) | 0.75 (0.59-0.95) | 0.89 (0.81-0.97) | 0.02 |

^a^ Model 1: adjusted for age (years, continuous), sex (male, female), race/ethnicity (non-Hispanic white, non-Hispanic black, Mexican American, others), secondhand smoking (yes, no).

^b^ Model 2: further adjusted for body mass index (< 25, 25-<30, ≥ 30 kg/m^2^), education attainment (under high school, high school, above high school), family poverty income ratio (< 1.3, 1.3-<3.5, ≥ 3.5), alcohol consumption (never, former, current), physical activity (never, moderate, vigorous), total energy intake (kcal, continuous), Healthy Eating Index-2015 score (continuous), urinary iodine (< 100, ≥ 100 μg/L), systolic blood pressure (mmHg, continuous), diastolic blood pressure (mmHg, continuous), and antihypertensive drug treatment (yes, no).

^d^ Per 1 μg/g creatinine increment in log-transformed urinary thiocyanate.

Abbreviations: CVD, cardiovascular disease; HR, hazard ratio.

## Supplementary Table 10. Associations between urinary thiocyanate and risks of cardiovascular disease and all-cause mortality among non-smoking adults, after applying multiple imputation under the missing-at-random to generate imputed datasets for missing covariates.

|  | **Quartiles of urinary thiocyanate** | | | | **Continuous^c^** | ***P*-value for trend** |
| --- | --- | --- | --- | --- | --- | --- |
|  | **1 (*n*=2623)** | **2 (*n*=2624)** | **3 (*n*=2620)** | **4 (*n*=2622)** |  |  |
| Range, mg/g | ≤ 0.50 | 0.50-0.89 | 0.89-1.51 | > 1.51 |  |  |
| CVD mortality |  |  |  |  |  |  |
| No. of death | 46 | 28 | 37 | 25 |  |  |
| Model 1^a^ | 1.00 (reference) | 0.71 (0.42-1.22) | 0.89 (0.57-1.40) | 0.51 (0.30-0.87) | 0.77 (0.64-0.92) | 0.02 |
| Model 2^b^ | 1.00 (reference) | 0.79 (0.49-1.27) | 1.01 (0.65-1.57) | 0.58 (0.35-0.96) | 0.80 (0.67-0.95) | 0.08 |
| All-cause mortality |  |  |  |  |  |  |
| No. of death | 262 | 166 | 187 | 185 |  |  |
| Model 1^a^ | 1.00 (reference) | 0.78 (0.61-0.99) | 0.83 (0.68-1.02) | 0.70 (0.57-0.86) | 0.86 (0.80-0.94) | 0.001 |
| Model 2^b^ | 1.00 (reference) | 0.80 (0.66-0.98) | 0.90 (0.74-1.09) | 0.80 (0.66-0.97) | 0.90 (0.83-0.96) | 0.04 |

^a^ Model 1: adjusted for age (years, continuous), sex (male, female), race/ethnicity (non-Hispanic white, non-Hispanic black, Mexican American, others), secondhand smoking (yes, no).

^b^ Model 2: further adjusted for body mass index (< 25, 25-<30, ≥ 30 kg/m^2^), education attainment (under high school, high school, above high school), family poverty income ratio (< 1.3, 1.3-<3.5, ≥ 3.5), alcohol consumption (never, former, current), physical activity (never, moderate, vigorous), total energy intake (kcal, continuous), dietary fiber (g, continuous), *β*-carotene (mg, continuous), folate (μg, continuous), vitamin K (μg, continuous), total fruits score (continuous), total dairy score (continuous), whole grains score (continuous), urinary iodine (< 100, ≥ 100 μg/L), and hypertension (yes, no).

^c^ Per 1 μg/g creatinine increment in log-transformed urinary thiocyanate.

Abbreviations: CVD, cardiovascular disease; HR, hazard ratio

## Supplementary Figure 1. Flow diagram of participants selectionin National Health and Nutrition Examination Survey 2001-2014.


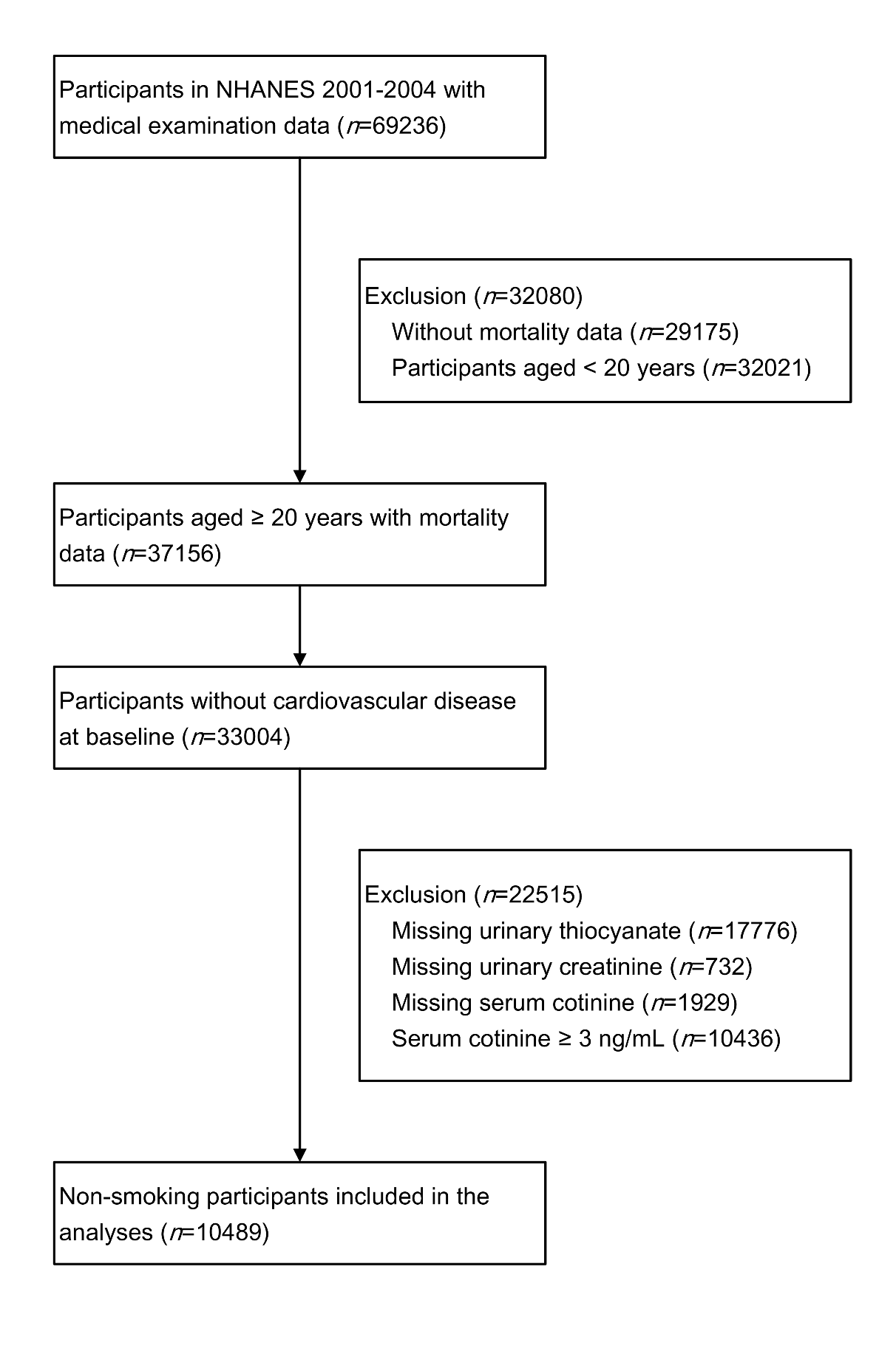


Abbreviation: NHANES, National Health and Nutrition Examination Survey.

## Supplementary Figure 2. Adjusted hazard ratios for cardiovascular disease mortality associated with each 1 μg/g creatinine increment in log-transformed urinary thiocyanate in subgroups defined by age, sex, race/ethnicity, obesity, secondhand smoking, current drinking, hypertension, and diet quality among non-smokers.


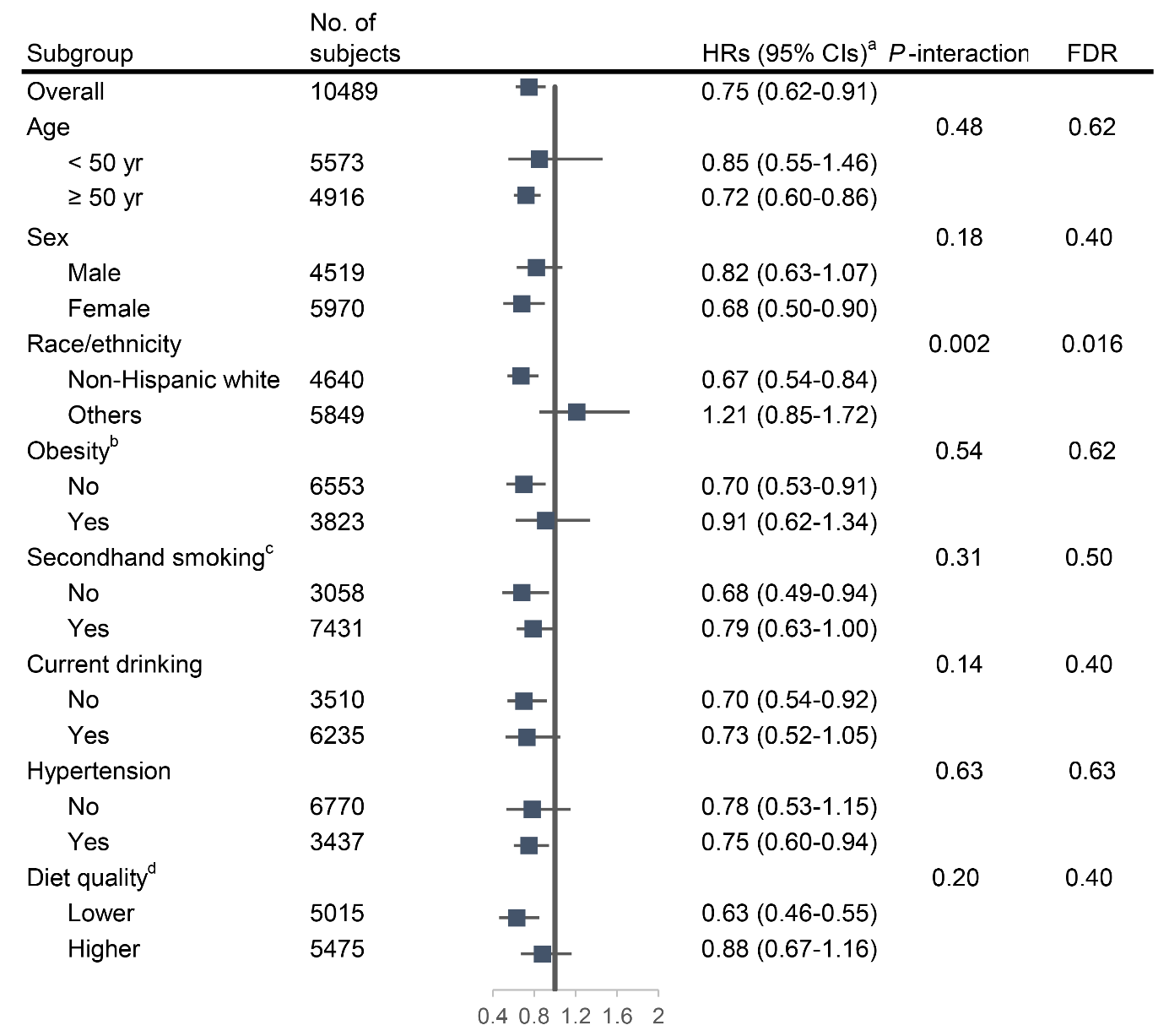


^a^ Adjusted for age (years, continuous), sex (male, female), race/ethnicity (non-Hispanic white, non-Hispanic black, Mexican American, others), secondhand smoking (yes, no), body mass index (< 25, 25-<30, ≥ 30 kg/m^2^), education attainment (under high school, high school, above high school), family poverty income ratio (< 1.3, 1.3-<3.5, ≥ 3.5), alcohol consumption (never, former, current), physical activity (never, moderate, vigorous), total energy intake (kcal, continuous), Healthy Eating Index-2015 score (continuous), urinary iodine (< 100, ≥ 100 μg/L), and hypertension (yes, no).

^b^ Obesity was defined as body mass index ≥ 30 kg/m^2^.

^c^ Secondhand smoking was defined as serum cotinine between 0.015 ng/mL and 3 ng/mL.

^d^ Lower diet quality was defined as a healthy eating index score less than median score and higher diet quality was defined as a score equal to or greater than the median score.

Abbreviation: FDR, false discovery rate; HR, hazard ratio.

## Supplementary References

1. Hung HC, Joshipura KJ, Jiang R, Hu FB, Hunter D, Smith-Warner SA, et al. Fruit and Vegetable Intake and Risk of Major Chronic Disease. *J Natl Cancer Inst* (2004) 96(21):1577-84. doi: 10.1093/jnci/djh296.

2. Zhang X, Shu XO, Xiang YB, Yang G, Li H, Gao J, et al. Cruciferous Vegetable Consumption Is Associated with a Reduced Risk of Total and Cardiovascular Disease Mortality. *Am J Clin Nutr* (2011) 94(1):240-6. doi: 10.3945/ajcn.110.009340.

3. Blekkenhorst LC, Bondonno CP, Lewis JR, Devine A, Zhu K, Lim WH, et al. Cruciferous and Allium Vegetable Intakes Are Inversely Associated with 15-Year Atherosclerotic Vascular Disease Deaths in Older Adult Women. *J Am Heart Assoc* (2017) 6(10):e006558. doi: 10.1161/jaha.117.006558.

4. Yu D, Zhang X, Gao YT, Li H, Yang G, Huang J, et al. Fruit and Vegetable Intake and Risk of Chd: Results from Prospective Cohort Studies of Chinese Adults in Shanghai. *Br J Nutr* (2014) 111(2):353-62. doi: 10.1017/s0007114513002328.

5. Bhupathiraju SN, Wedick NM, Pan A, Manson JE, Rexrode KM, Willett WC, et al. Quantity and Variety in Fruit and Vegetable Intake and Risk of Coronary Heart Disease. *Am J Clin Nutr* (2013) 98(6):1514-23. doi: 10.3945/ajcn.113.066381.

6. Joshipura KJ, Ascherio A, Manson JE, Stampfer MJ, Rimm EB, Speizer FE, et al. Fruit and Vegetable Intake in Relation to Risk of Ischemic Stroke. *JAMA* (1999) 282(13):1233-9. doi: 10.1001/jama.282.13.1233.

7. Joshipura KJ, Hung HC, Li TY, Hu FB, Rimm EB, Stampfer MJ, et al. Intakes of Fruits, Vegetables and Carbohydrate and the Risk of Cvd. *Public Health Nutr* (2009) 12(1):115-21. doi: 10.1017/s1368980008002036.

8. Johnsen SP, Overvad K, Stripp C, Tjønneland A, Husted SE, Sørensen HT. Intake of Fruit and Vegetables and the Risk of Ischemic Stroke in a Cohort of Danish Men and Women. *Am J Clin Nutr* (2003) 78(1):57-64. doi: 10.1093/ajcn/78.1.57.

9. Mizrahi A, Knekt P, Montonen J, Laaksonen MA, Heliövaara M, Järvinen R. Plant Foods and the Risk of Cerebrovascular Diseases: A Potential Protection of Fruit Consumption. *Br J Nutr* (2009) 102(7):1075-83. doi: 10.1017/s0007114509359097.

10. Larsson SC, Virtamo J, Wolk A. Total and Specific Fruit and Vegetable Consumption and Risk of Stroke: A Prospective Study. *Atherosclerosis* (2013) 227(1):147-52. doi: 10.1016/j.atherosclerosis.2012.12.022.

11. Wang JB, Fan JH, Dawsey SM, Sinha R, Freedman ND, Taylor PR, et al. Dietary Components and Risk of Total, Cancer and Cardiovascular Disease Mortality in the Linxian Nutrition Intervention Trials Cohort in China. *Sci Rep* (2016) 6:22619. doi: 10.1038/srep22619.

12. Lockheart MS, Steffen LM, Rebnord HM, Fimreite RL, Ringstad J, Thelle DS, et al. Dietary Patterns, Food Groups and Myocardial Infarction: A Case-Control Study. *Br J Nutr* (2007) 98(2):380-7. doi: 10.1017/s0007114507701654.
